# A Possible Phenotype-to-Genotype Association of Novel Single-Nucleotide Variants in the Coding Exons of the *ZNF469* Gene to Arterial Aneurysmal and Dissection Diseases

**DOI:** 10.3390/ijms252413730

**Published:** 2024-12-23

**Authors:** Adam Wolf, Peyton Moore, Charles Hong, Mohanakrishnan Sathyamoorthy

**Affiliations:** 1 Sathyamoorthy Laboratory, Department of Medicine, Burnett School of Medicine at TCU, Fort Worth, TX 76104, USA; 2 Department of Medicine, College of Human Medicine, Michigan State University, East Lansing, MI 48824, USA; 3 Consultants in Cardiovascular Medicine and Science, Fort Worth, TX 76104, USA; 4 Fort Worth Institute for Molecular Medicine and Genomics Research, Fort Worth, TX 76104, USA

**Keywords:** *ZNF469*, collagen matrix proteins, aneurysm, vertebral artery dissection, genetic basis of disease

## Abstract

After reporting the first known clinical case associating compound heterozygous single-nucleotide variants in Exon 2 of *ZNF469* to aortic aneurysmal and iliac dissection, we began prospective surveillance in our vascular genetic practice for similar cases. Herein, we present nine (9) subjects from a total cohort of 135 with arterial aneurysms or dissections who revealed single-nucleotide variants in *ZNF469* with no other alterations in a panel of 35 genes associated with aneurysmal and dissection disorders. Five out of nine (5/9) single-nucleotide variants were in Exon 1, and four out of nine (4/9) mutations were in Exon 2, both of which are principal coding exons for this gene. Eight out of nine (8/9) were ACMG variants of unknown significance (VUSs), and one out of nine (1/9) was an ACMG pathogenic mutation previously associated to brittle cornea syndrome (BCS). Of our nine subjects, four (44.4%) experienced clinically significant vascular dissection, and four (44.4%) had a family history of one or more first-degree relatives with aneurysmal or dissection diseases. This novel genetic case series significantly strengthens our initial discovery of *ZNF469’s* potential association with arterial aneurysmal/dissection diseases through the study of this cohort of unrelated patients.

## 1. Introduction

### 1.1. The Extracellular Matrix (ECM)

The extracellular matrix is composed of co-existing macromolecules designed to carry out many dynamic functions [1]. Due to its important role in tissue structure and function, any dysfunction may produce catastrophic consequences. Elastin, collagen, proteoglycans, and glycoproteins are the major contributors to the formation of the ECM and allow the matrix to have a wide variety of biochemical and physical properties [1]. The variety of components also allow the ECM to help carry out cellular functions, including migration, polarity, differentiation, proliferation, and apoptosis [2]. In the vascular system of humans, the tunica media plays a significant role in the structural and mechanical properties of the vessel. Further, the tunica adventitia, the outermost layer, is rich in collagen, allowing it to resist arterial-pressure-induced vascular rupture. Signals originating from within the adventitia play essential roles in the regulation of vascular development, wall remodeling, and vascular disease [3]. Due to the role that the ECM component collagen plays in maintaining the structure of the adventitial layer, ECM integrity and proper function is vital for the proper strength and function of the tunica adventitia [4].

The pathogenesis of many vascular diseases involves disruptions in the ECM. Pathogenesis in these diseases is typically through physiologic responses to injury (hypertension-induced remodeling) as well as ECM dysfunction (cystic medial necrosis leading to aortic aneurysm development) [5]. The complications of ECM dysfunction which result in aneurysmal disease may lead to spontaneous dissection. Arterial dissections arise as a tear in the tunica intima, which allows a high-pressure flow into the intimal–medial space. This disruption of the flow allows for communication between the true and false lumen [6]. Studies suggest that up to 40% of dissection patients will develop a post-dissection aneurysm [7]. 

The literature has illustrated that mutations in 12 specific ECM genes are pathogenic and syndromic in thoracic aortic aneurysm and dissection (TAAD). These genes include *FBN1*, *COL3A1*, *COL1A2*, *COL5A1*, *COL5A2*, *LOX*, *MFAP5*, *EMILIN1*, *ELN*, *BGN*, and *EFEMP2* [8]. A single gene being the cause of a non-syndromic TAAD has previously been established, as 35 genes are routinely tested for in the genetic screening for familial TAAD risk stratification [8,9,10]. A further understanding of the genetic contributors of TAAD will enhance screening tools and allow for a more accurate calculation of the risk for patients and their family members. 

### 1.2. ZNF469—A Regulatory Gene for the Extracellular Matrix

*ZNF469* is a zinc finger protein-encoding gene, with limited characterization to date, located on chromosome 16q24.2, which encodes a collagen-related protein product called zinc finger protein 469 [OMIM ID 612078, HGNC ID 2316, NCBI Gene 84627, and Genomic coordinates (GRCh38): 16:88,100,931-88,440,753 (from NCBI)]. Given the 30% sequence homology to collagens I and III, and Clusterin, there is a strongly suggested role in the assembly or organization of collagen fibers [11]. Prior to our group reporting the first association with arterial aneurysmal diseases [12], mutations in this gene were only reported in brittle cornea syndrome, an ocular connective tissue disease [13,14,15,16]. Though the exon structure has been debated, it is currently recognized as a five-exon gene [16,17]. *ZNF469* belongs to the zinc finger protein family of genes, and many ZNF proteins are known to be involved in vascular development and pathogenesis [18]. In a murine study involving knockout models, a loss-of-function mutation in the gene led to a decrease in the corneal biomechanical strength, likely due to impacts on the ECM [19]. Very recent work by Aryiachet et al. demonstrated that the knockdown of *ZNF469* in hepatic stellate cells by a shRNA lentiviral approach led to decreased collagen production, and the over-expression of *ZNF469* by the CRISPRa methodology led to enhanced collagen production [20]. The vascular ECM is vital for structure and function, and induced mutations in *ZNF469* have altered the production of ECM components, including collagens I and III, Thrombospondin-1 (THBS1), and Clusterin [11,16]. 

The disruption in these ECM components due to variants in *ZNF469* also disrupt the integrity of the ECM. Collagen is a vital constituent of the vasculature [21]. Collagens I and II, accounting for roughly 90% of arterial collagen, have been implicated in multiple disorders, including vascular Ehlers Danlos syndrome (vEDS) and Osteogenesis Imperfecta [21,22,23]. Current research suggests that *ZNF469* variants may result in the dysregulation of collagen through various mechanisms, including the downregulation of *COL4A1* and *COL11A1*, the dysfunction of collagen receptors, and through the thinning of collagen fibrils [16,24,25]. 

Thrombospondin-1, a glycoprotein known to interact with ECM components and a potent inhibitor of angiogenesis, also interacts with *ZNF469.* The role of *TMBSP1* in blocking angiogenesis involves the upregulation of endothelial cell pro-apoptotic pathways, as well as the antagonization of the vascular endothelial growth factor (VEGF). Murine models have demonstrated that maladaptive changes in *TMBSP1* signaling play a crucial role in TAA formation through cytoskeleton remodeling disruption, as well as the disruption of elastin [26].

Clusterin, a glycoprotein with known diverse functions, is involved in the pathogenesis of a wide spectrum of diseases. The glycoprotein is illustrated to play a crucial role in vascular smooth muscle cell (VSMC) differentiation, phenotype modulation, and VSMC nodule formation [27]. Neointimal hyperplasia after a vascular insult is seen in Clusterin-deficient murine models due to the inhibition of VSMC proliferation and VSMC cell cycle arrest [28]. Due to the crucial interactions that *ZNF469* has with vital ECM components, the role of *ZNF469* variants in TAAD development requires further investigation. 

This study, derived from genetic analysis of 135 subjects, is the first to report novel variants in the extracellular matrix regulatory gene, *ZNF469* in a cohort of eight non-syndromic cases and a potential novel syndromic case of aortic aneurysms and/or arterial dissections among unrelated patients.

## 2. Results

### 2.1. Single-Nucleotide Variants Identified in Exon 1

#### 2.1.1. Subject 1—p.T910I: ACGM Classification Variant of Unknown Significance

A 37-year-old Caucasian American male with a bicuspid aortic valve (AV) (11 o’clock and 4 o’clock orientation), with no aortic insufficiency (AI) or stenosis (AS) by echocardiography, whose maternal uncle developed an ascending aortic aneurysm at the age of 63. Given this patient’s bicuspid valve and a family history significant for aortic aneurysm, we completed a CT angiogram (CTA), demonstrating an ascending aorta measuring 3.4 cm in diameter, and offered him genetic testing to guide long-term management. This revealed a heterozygous missense mutation VUS of p.T910I in the *ZNF469* gene with a Grantham Score (GS) of 89 (a similar amino acid substitution) in a position that contains Isoleucine in other vertebrates as a reference. No other mutations in the remaining 34 genes were discovered. The variant p.t910I (also known as c.2729C>T) replaces an alpha-indifferent/beta-former with an alpha-former/strong beta-former. Our analysis of the GnomAD or ClinVar databases predicts this mutation to be tolerated; however, the clinical significance for this case remains compelling and requires more direct biologic investigation [29,30].

#### 2.1.2. Subject 2—p.G1933V: ACGM Classification Variant of Unknown Significance 

**Figure 1:**
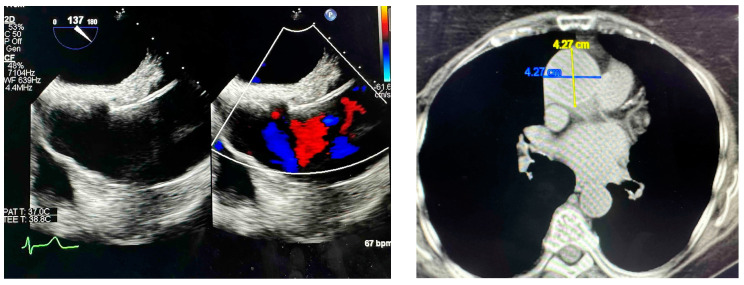
(**Left Panel**): TEE imaging demonstrating a 4.3 cm ascending aortic aneurysm in subject 2. (**Right Panel**): CTA measuring an ascending aortic aneurysm of 4.3 cm × 4.3 cm in subject 2.

A 76-year-old Caucasian American female was diagnosed by TEE during an aortic valve evaluation with an aortic aneurysm measuring 4.3–4.4 cm (Figure 1), who subsequently underwent genetic testing, which revealed a heterozygous mutation VUS of p.G1933V in *ZNF469*, with a GS of 109 (a dissimilar amnio acid substitution), in a position that is poorly conserved in vertebrates. The p.G1933V variant, also known as c.5798G>T, replaces a strong alpha-breaker/beta-breaker with an alpha-former/strong beta-former. This heterozygous mutation was not present in the GnomAD or ClinVar databases, so the clinical significance of this variant is of potential biologic relevance, thus requiring more direct study. No other mutations in the remaining 34 genes within the TAAD sequencing panel were found in this subject.

#### 2.1.3. Subject 3—p.V271D: ACGM Classification Variant of Unknown Significance

A 45-year-old Caucasian American female with a history of bilateral vertebral artery dissections following a neck injury identified by magnetic resonance angiography during hospitalization for her symptomatic presentation. The patient’s mother expired from a ruptured cerebral artery aneurysm at the age of 55, and her son was diagnosed with Ehlers Danlos syndrome. Genetic testing revealed a p.V271D heterozygous mutation VUS in *ZNF469*, with a GS of 152. Of the 35 genes in the TAAD panel that were assessed, no other mutations were found. This mutation results in a change from valine to aspartic acid at codon 271, which have highly dissimilar properties. The p.V271D variant (also known as c.812T>A) replaces an alpha-former/strong beta-former with a weak alpha-former/strong beta-breaker. Our analysis of the GnomAD or ClinVar databases predicts this mutation to be tolerated; however, the clinical significance for this case remains compelling and requires more direct biologic investigation [29,30]. 

#### 2.1.4. Subject 4—C.2193delG: ACGM Classification Variant of Unknown Significance

A 25-year-old Caucasian American male presented in 2021 for cardiovascular risk stratification of connective tissue disease. The patient had a marfanoid appearance, including a height of 6′5″, a weight of 160 lbs., pectus excavatum, and hypermobility/laxity of multiple joints. His echocardiogram demonstrated a normal-appearing ascending aorta with a bicuspid AV angulated at 3 o’clock and 9 o’clock without AI or AS. Given the patient’s presentation, we offered genetic testing, which revealed a C.2193delG mutation in the *ZNF469* gene, a mutation in Exon 1 that causes a frameshift resulting in a premature stop codon (p.T732Pfs*68). This was the only genetic mutation found in the patient among the entire 35-gene panel. A CTA of his chest demonstrated fusiform ectasia of the proximal descending thoracic aorta without dilatation. His mutation was considered pathogenic for brittle cornea syndrome, for which he had undergone a corneal ophthalmology consultation. Given the pathogenic nature of this mutation, the clinical significance for this case is compelling, and more direct biologic investigation is planned, along with long-term longitudinal clinical assessments and aortic imaging.

#### 2.1.5. Subject 5—p.A1001D: ACGM Classification Variant of Unknown Significance

A 60-year-old Caucasian female presented to our program in 2023 with a stunning history of arterial dissection diseases. In 2004, she was diagnosed with an acute abdominal aortic dissection extending into the mesenteric artery and underwent urgent operative intervention. In 2014, she presented to an emergency department with unstable angina and was diagnosed with an acute ST elevation MI. Urgent cardiac catheterization revealed an acute spontaneous coronary artery dissection. She did not have a history of hypermobility, cartilaginous concerns, and had no history of corneal disease. Given this history, we proceeded with clinical genetic testing, which revealed a single-nucleotide variant, c.3002C>G, located in the coding Exon 1 of the *ZNF469* gene, which results from a C to A substitution at nucleotide position 3002. The p.A1001D mutation results in an alanine, which is neutral and non-polar at codon 1001, being replaced by aspartic acid, an amino acid that is acidic and polar, making the two amino acids dissimilar. No other variants in the remaining 34 genes in the TAAD panel were found [29,30].

### 2.2. Single-Nucleotide Variants Identified in Exon 2

#### 2.2.1. Subject 6—p.R1875C: ACGM Classification Variant of Unknown Significance

A 61-year-old Caucasian American female with no prior cardiac history or known risk factors experienced a spontaneous coronary artery dissection (SCAD) of the left anterior descending artery in 2014. Genetic testing was offered due to her personal history of SCAD and a similar history in her biological sister. The results of this genetic testing demonstrated a p.R1875C variant in the *ZNF469* gene, a variant with a minor allele frequency (MAF) of 0.018%. The Grantham Score of 180 revealed an amino acid substitution between dissimilar amino acids. This variant, also known as c5623C>T, replaces an alpha/beta-indifferent amino acid with an alpha-indifferent/beta-former. This sequence change replaces arginine, a basic and polar amino acid, with cysteine, a neutral and slightly polar amino acid, at codon 1875. This change appears to be conserved across numerous mammalian species. Our analysis of the GnomAD or ClinVar databases predicts this mutation to be tolerated; however, the clinical significance for this case remains compelling and requires more direct biologic investigation [29,30]. No other variants were isolated in this subject.

#### 2.2.2. Subject 7—p.Q3094R, p.G2871S, and P. S2637T: ACGM Classification Variant of Unknown Significance

**Figure 2:**
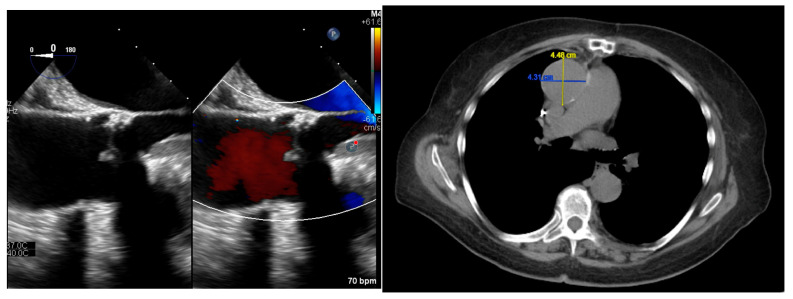
(**Left Panel**): TEE imaging at the omniplane of zero degrees, demonstrating a 4.5 cm ascending aortic aneurysm in subject 7—note the aortic dilation around the existing bioprosthetic aortic valve. (**Right Panel**): Aortic CTA demonstrating the 4.5 cm ascending aortic aneurysm in subject 7.

A 72-year-old African American female developed chronic, bilateral distal aortoiliac dissections that were repaired with endovascular stenting in our program, and, in subsequent years, underwent a TEE that demonstrated the enlargement of the ascending aorta to 4.5 cm (Figure 2). We learned of a significant history of unexplained aortic diseases and dissection in her extended family, allowing us to generate a family pedigree [12]. Subsequent genetic testing revealed the following three heterozygous mutations in Exon 2 of the *ZNF469* gene: p.Q3094R, p.G2871S, and p.S2637T. The variants in the *ZNF469*, p.G2871S and p.S2637T, produce GSs of 56.00 (a similar amnio acid substitution and an alpha-indifferent/beta-breaker to alpha-indifferent/beta-former) and 58.00 (a similar amnio acid substitution and a strong alpha-breaker/beta-breaker to alpha-indifferent/beta-breaker), respectively, while the p.Q3094R substitution yields a GS of 43.00 (a highly similar amnio acid substitution and an alpha-/beta-former to alpha-/beta-indifferent). The p.Q3094R variant (also known as c.9281A) replaces a neutral and polar amino acid in glutamine with arginine, a basic and polar amino acid. This variant is suggested to be tolerated by in silico analysis, and the clinical significance is unknown [29,30]. The p.G2871S variant (also known as c.8611G>A) replaces a neutral and non-polar glycine with serine, a neutral and polar amino acid. This missense mutation is predicted to be tolerated by in silico analysis and is classified as a likely benign mutation [29,30]. The p.S2637T variant (also known as c.7909T>A) replaces serine, a neutral and polar amino acid, with threonine, a neutral and polar amino acid, at codon 2637. This amino acid position is not well conserved in vertebrate species [29,30]. No other variants were discovered in the tested TAAD panel of genes, and we previously reported this compound heterozygote case as the first genotype–phenotype association of *ZNF469* to vascular aneurysmal disease [12]. The strong multi-generation prevalence of aortic disorders within the family of this proband appears to have Mendelian characteristics with incomplete penetrance, and further study of this familial cohort is underway in our program.

#### 2.2.3. Subject 8—p.s2646F: ACGM Classification Variant of Unknown Significance

A 56-year-old Asian American female with a history of familial aneurysmal disease presented to our clinic. The family history includes her mother having developed an ascending aortic aneurysm, which was found on imaging at the age of 78, her maternal aunt expiring due to the complications of a vertebral artery aneurysm rupture at the age of 40, and several other family members with a history of aneurysms. Due to this extensive family history, we ordered a CTA, which revealed her index aortic diameter to be 3.3 cm, and proceeded with genetic testing. This testing revealed a VUS p.S2646F variant (c.7937C>T), which replaces the alpha/beta-breaker with an alpha-former/strong beta-former. This altered sequence replaces serine, a neutral and polar amino acid, with phenylalanine, a neutral and non-polar amino acid, at codon 2646. These amino acids have highly dissimilar properties, and the amino acid position is poorly conserved in vertebrate species. Our analysis of the GnomAD or ClinVar databases predicts this mutation to be tolerated; however, the clinical significance for this case remains compelling and requires more direct biologic investigation [29,30]. This was the only positive variant within the TAAD panel of 35 genes for this subject.

#### 2.2.4. Subject 9—p.A3542V: ACGM Classification Variant of Unknown Significance

**Figure 3:**
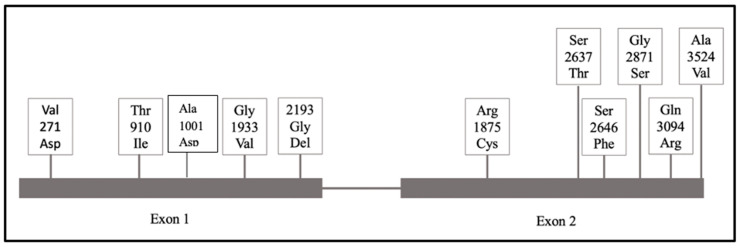
Approximate positions of all mutations across this cohort. The mutations observed in this cohort are confined to the transcriptional coding Exons 1 and 2, which, through the subsequent regulatory effects on the target genes collagen 1 (COL1A1), collagen 3 (COL3A1), Clusterin (CLU), and Thrombospondin-1 (THBS1), lead to structural alterations in the extracellular matrix, thereby creating our observed phenotypes. Furthermore, 3/6 variants in Exon 1 and 5/6 variants in Exon 2 reside in close genomic proximity, suggesting biologic relevance to these regions.

An 83-year-old Caucasian American male presented with a history of a 4.5 cm aortic aneurysm and celiac artery aneurysm with stable dissection. Genetic testing was performed, which revealed a heterozygous mutation VUS p.A3542V (poorly conserved position in vertebrates) in *ZNF469*. The p.A3542V variant, also known as c.10625 C>T, had a GS of 64 (a similar amnio acid substitution), replacing a strong alpha-former/beta-indifferent amino acid with an alpha-former/strong beta-former [29,30]. No other variants were isolated in this subject within the TAAD panel of genes. This variant replaces alanine, a neutral and non-polar AA, with valine, a neutral and non-polar AA, which are two amino acids with similar properties. The valine amino acid residue is found in multiple mammalian species, making it unlikely that protein function is negatively affected. Our analysis of the GnomAD or ClinVar databases predicts this mutation to be tolerated; however, the clinical significance for this case remains compelling and requires more direct biologic investigation [29,30]. Table 1 summarizes the subject data and Figure 3 illustrates the approximate genomic positions of these single-nucleotide variants along Exon 1 and Exon 2.

## 3. Materials and Methods

### 3.1. Clinical Genetic Testing

From a cohort of patients with aneurysm/dissection disorders who underwent clinical genetic testing at CCMS-FW (*n* = 135) to guide clinical decision making, we identified nine patients with mutations in *ZNF469*. After receiving human subject study approval from the TCU (IRB#2022-106), we consented, interviewed, and performed a detailed EMR chart review of each of these 9 subjects who possessed a variant in *ZNF469* at Consultants in Cardiovascular Medicine–Fort Worth PLLC (CCMS-FW) between April 2022 and May 2023. All of the patient related records were kept on file at CCMS-FW, in compliance with the TCU IRB and U.S. Federal HIPPA requirements.

All of the subjects underwent clinical genetic testing using a commercially available 35 TAAD-gene panel supplied and analyzed by Ambry Genetics, Aliso Viejo, CA, USA. utilizing genomic deoxyribonucleic acid-isolated samples obtained via saliva. This gene panel targets the detection of DNA sequence mutations in 35 genes “(*ACTA2*, *BGN*, *CBS*, *CHST14*, *COL1A1*, *COL1A2*, *COL3A1*, *COL5A1*, *COL5A2*, *EFEMP2*, *FBN1*, *FBN2*, *FKBP14*, *FLNA*, *FOXE3*, *LOX*, *MAT2A*, *MED12*, *MFAP5*, *MYH11*, *MYLK*, *NOTCH1*, *PLOD1*, *PRDM5*, *PRKG1*, *SKI*, *SLC2A10*, *SMAD3*, *SMAD4*, *TGFB2*, *TGFB3*, *TGFBR1*, *TGFBR2*, *TNXB* (excluding Exons 32–44), and *ZNF469*) by either the Next-Generation or Sanger sequencing of all coding domains, and well into the flanking 5′ and 3′ ends, of all of the introns and untranslated regions” [31,32,33,34,35,36,37]. “Gross deletion/duplication analysis determines the gene copy number for the covered exons and untranslated regions of all genes (excluding *CBS* and *TNXB* Exons 32–44) [31,32,33,34,35,36,37]”. “Bait capture methods were utilized for the enrichment of coding exon sequences of interest using biotinylated oligonucleotide probes, and the subsequent polymerase chain reaction and sequencing, utilizing NCBI reference sequences”. “Additional Sanger sequencing was performed for any regions missing or with any insufficient read-depth coverage for reliable heterozygous variant detection. Based on the read depth from the NGS data, followed by a confirmatory orthogonal method, as needed, the sequence analysis for *ZNF469* was based on the NCBI reference sequences for *ZNF469:* NM_001127464.1”. The sequencing platform utilized, the depth of sequencing, and the quality control measures are proprietary to the commercial entity utilized for genetic testing; these are likely industry standard Illumina platforms, conducted at a 30X depth, and with the stringent quality control required for CLIA certification. It is important to note that these 9 subjects demonstrated mutations only in ZNF469 without alterations in any of the other 34 genes sequenced. Once the results were received and reviewed, genetic counseling was provided on a 1:1 basis with each patient at CCMS-FW.

### 3.2. Statistics

Statistical analyses were not utilized in this study due to the small sample size of (*n =* 9). This case series is designed to report on variants in the *ZNF469* gene associated with TAAD phenotypes. Future studies of larger sample sizes may be successful in comparing the differences in the age of the subjects to the localization of the mutations on either Exon 1 or Exon 2. 

### 3.3. Single-Nucleotide Variant (SNV) Analytic Methods

A detailed analysis of SNVs was performed using standard methodology within both the genome aggregation database (GnomAD) (https://gnomad.broadinstitute.org, accessed on 11 August 2023) and ClinVar (https://www.ncbi.nlm.nih.gov/clinvar/, accessed on 11 August 2023) database; then, we reviewed any previously reported data if present for each SNV. The predicted amino acid properties, along with this in silico analysis, were used to establish our prediction on the biologic significance of each SNV. The GnomAD database provides data on genetic variation in human populations. Each variant in *ZNF469* was located in the database, and any existing data on the site were used. Similarly, ClinVar is a public database with genomic variation data, notably providing information on the potential clinical significance. Each variant, if present, was located in the database and reported.

### 3.4. Variant of Unknown Significance (VUS) Interpretation

The American College of Medical Genetics and Genomics (ACMG) recommendations and guidelines help to stratify variants into five categories based on likely pathogenicity. These categories include ‘pathogenic’, ‘likely pathogenic’, ‘uncertain significance’, ‘likely benign’, and ‘benign.’ The criteria used to determine the pathogenicity include evidence such as population data, computational data, and functional data [38]. The commercial laboratory used to compile the data in each of these cases strictly follows ACGM guidelines to determine the pathogenicity. The evidence used by this CLIA-certified laboratory to stratify variants includes clinical data, the molecular impact, functional assays, and population frequencies. Each variant was scored and placed into the respective categories based on points. The point criteria include ≤4 (benign) to 2 (likely benign), 1 to +5 (uncertain significance), +6 to +9 (likely pathogenic), and ≥10 (pathogenic) [39]. Interpretation was provided to the research team after the collection, and analysis was then performed.

### 3.5. ClinVar and Gnom AD Frequency of Variant Analysis

The frequency of each variant has not been reported in public databases, as each of the described VUSs are novel and unreported to date. We performed queries for each variant in the ClinVar and GnomAD databases [29,30], which predicted the likelihood of tolerance in silico for each individual mutation. Though in silico analysis is a useful predictive tool, it is not definitive in establishing the overall effect of a variant, which would require definitive experimental confirmation. The continued collection of data is required to assess the population frequency, and, as others report, through additional study, the variants in *ZNF469*, the population frequency, and the elucidation for the VUS pathogenicity will change.

## 4. Discussion

This genetic case series is an extension of our initial case report, which was the first to demonstrate an association between variants in the *ZNF469* gene and the development of arterial aneurysmal disease [12]. Prior to this case series and the initial case report, variants in *ZNF469* were only reported to be associated with the ocular disease brittle cornea syndrome (BCS). BCS, a severe ocular disease primarily characterized by corneal thinning, has also been associated with another ECM regulatory gene, *PRDM5.* Consistent with this report, the altered expression of other ECM-associated genes was observed in patients with BCS, particularly in those with variants in *ZNF469* [16]. Despite *ZNF469*’s role as a vital gene to maintain the ECM structure and appropriate function, no prior studies have reported associations between variants in the gene and the development of arterial aneurysmal disease. 

Prior linkage between *ZNF469* and BCS, paired with our association of this gene with arterial aneurysmal disease, teases a potential association between an ECM-related disease state in the human eye and vascular system [40]. This plausible connection would likely be secondary to similarities in the ocular and vascular ECMs, consisting of many shared cellular and acellular components. Further, both are heavily dependent on collagen for their structure and function [41]. Due to these similarities, it appears likely that a gene responsible for ECM regulatory functions, such as *ZNF469*, may play a role in disease pathogenesis at both sites [19,24]. Our laboratory is currently investigating this potential association [40].

Variants in other ECM genes have also been associated with isolated arterial aneurysmal disease in the past. Notable genes include the *FBN1*, *LOX*, *MYH11*, *ACTA2*, *MYLK*, *PRKG1*, *COL3A1*, *TGFBR2*, *TGFBR1*, *TGF-β2*, and *SMAD3* genes [42]. Although the pathogenesis depends on the gene in question, disruptions in ECM integrity and function underlie aneurysmal formation when each of these genes’ protein products are disrupted. This prior knowledge of the disruption of other ECM genes resulting in aneurysm formation increases our suspicion for *ZNF469* being a potential pathogenic variant as well. 

Pathogenic variants in other ECM genes are known to be responsible for the development of various ECM syndromic diseases such as EDS and Marfan Syndrome. Potential syndromic disease was evident in subject 4, whose *ZNF469* variant was a known pathogenic mutation for BCS. Despite having this known variant, the patient also notably had a marfanoid body habitus and fusiform ectasia of his aorta, somewhat resembling an EDS clinical picture which is typically associated with variants in *COL* genes. Prior literature has demonstrated the relationship between *ZNF469* and *COL3A1*, increasing our suspicion that the variant *ZNF469* may negatively impact the downstream signaling to *COL3A1*, impacting collagen production and causing an EDS-like phenotype. Our suspicion for a syndromic disease is increased in this patient, particularly due to his ectatic aorta at the age of 25. 

When assessing the differences in patient cohorts between those with Exon 1 and Exon 2 mutations, there was an apparent difference in ages of nearly 20 years between the two groups (Table 1). The Exon 1 cohort was, on average, younger at the time of presentation, and contained personal and family histories of marfanoid habitus and multiple bicuspid aortic valves, in addition to aneurysmal and dissection disease. In contrast, the Exon 2 cohort displayed aneurysmal and dissection phenotypes, while lacking some of the potentially syndromic effects demonstrated in the Exon 1 cohort, as well as being older. These differences may provide us with insight into the importance that each exon plays in the structure and function of the *ZNF469* gene and its downstream regulatory effect on target genes and raises the question of the influence exon position and age in the development of these aneurysmal phenotypes. Although our limited sample size of *n =* 9 limits us from identifying a true statistical significance, this association warrants further investigation in the future with a larger cohort. 

## 5. Conclusions

Our initial report, and this broader study, expands the association of *ZNF469*, with its known effects on the ECM structure and function, to vascular aneurysmal- and dissection-related diseases. The association made in our cohort, given the strong personal and family histories for aneurysmal and dissection disease, implies that *ZNF469* may play a pivotal role in ECM regulation through its downstream regulatory effects on different collagens, Thrombospondin-1, or Clusterin. Our discovery warrants further investigation, which may ultimately define *ZNF469* as an ACMG pathogenic gene in aneurysmal diseases, which would alter the standard of care for screening and the management of patients who have strong personal and family histories of aortic and arterial aneurysmal diseases.

## 6. Limitations

This study establishes a potential association between *ZNF469* variants in aneurysmal and dissection disorders, building on our first single-subject report [12]. Definitive attribution will require extensive characterization of how, or if, these variants affect the expression or behavior of proteins encoded by the downstream targets of *ZNF469*, and how these alterations influence the collagen matrix in vivo, as well as the mechanistic interactions between *ZNF469* variants. However, we find it novel, hypothesis-generating, and reportable that nine unrelated subjects with definite aneurysmal and dissection phenotypes all share mutations in Exon 1 and Exon 2 of *ZNF469*, which has a definitive role in the ECM biology, and hope this report will motivate further confirmatory work. Further limitations include confounding factors for TAAD development, including hypertension, which is variable across our cohort, a lack of knowledge about the definitive downstream molecular impacts of variants, as previously acknowledged, and an unclear temporal relationship between variants, age, and the development of the listed phenotype. Though not all of the subjects included have personal history of aneurysm or dissection, given their mutation status, young age, and positive family history, we suspect that these patients who have yet to manifest aneurysms have a predisposition to developing TAAD and thus will undergo close clinical monitoring. Taking this into consideration strengthens the accepted understanding that not all single-nucleotide variants or polymorphisms encode aberrant proteins that create pathologies, but may do so through alterations in regulatory signaling through post-transcriptional, epigenetic, and other factors that may influence our observed genotype-to-phenotype relationship.

## 7. Future Investigations

As we strengthen this potential association through familial genotyping for each proband presented in this report, we hope to further define *ZNF469* as a potential causal, pathogenic gene for non-syndromic vascular aneurysmal disease. Our laboratory is in the process of conducting further confirmatory studies to assess the potential impact of variants in ZNF469, including the immunohistochemical characterization of aortic samples recently obtained from one subject during aortic surgery, pursuing mechanistic studies in animal model systems, and population-level genomic analyses. We anticipate that our current and future work will help to establish variants in *ZNF469* as ACGM pathogenic mutations, which will lead to earlier and more definitive clinical action in patients with these mutations who manifest vasculopathy, potentially mitigating the associated risks of morbidity and mortality.

## Figures and Tables

**Table 1:** Description of patients with *ZNF469* variants. Included in the table are the categories of age, ethnicity, family history, exon/codon location, in silico analysis results, and the corresponding vascular phenotype.

Subject	Age	Gender	Ethnicity	Family Hx	Variant	Exon/Codon	In Silico Analysis	Vascular Phenotype
1	37	Male	Caucasian	Aortic aneurysm	p.T910I	Exon 1 Codon 910	Tolerated	Bicuspid aortic valve Aortic tortuosity without enlargement
2	76	Female	Caucasian	Unknown	p.G1933V	Exon 1 Codon 1993	Uncertain	Aortic aneurysm
3	45	Female	Caucasian	Cerebral aneurysm	p.V271D	Exon 1 Codon 271	Tolerated	Bilateral vertebral artery dissection
4	25	Male	Caucasian	Marfanoid habitus	C.2193delG	Exon 1 Codon 2193	Pathogenic	Fusiform ectasia of aorta Bicuspid aortic valve Marfanoid habitus
5	60	Female	Caucasian	None	p.A1001D	Exon 1 Codon 1001	Uncertain	Spontaneous iliac artery dissection and spontaneous coronary artery dissection
6	61	Female	Caucasian	Spontaneous coronary artery dissection	p.R1875C	Exon: 2 Codon 1875	Tolerated	Spontaneous coronary artery dissection (SCAD)
7	72	Female	African	Cerebral aneurysm Aortic aneurysm Dissections	p.Q3094R p.G2871S p. S2637T	Exon: 2 Codons 2637, 2871, 3094	Tolerated	Bilateral aortoiliac dissections Aortic aneurysm
8	56	Female	Asian	Arterial aneurysms	p.S2646F	Exon: 2 Codon 2646	Tolerated	Fusiform aortic ectasia
9	83	Male	Caucasian	Unknown	p.A3542V	Exon: 2 Codon 3542	Tolerated	Aortic and celiac aneurysm/dissection

## Data Availability

The data presented in this study are available on request from the corresponding author. The data are not publicly available due to patient privacy considerations.
